# Cross-platform expression profiling demonstrates that SV40 small tumor antigen activates Notch, Hedgehog, and Wnt signaling in human cells

**DOI:** 10.1186/1471-2407-6-54

**Published:** 2006-03-07

**Authors:** Mohamed Ali-Seyed, Noelani Laycock, Suresh Karanam, Wenming Xiao, Eric T Blair, Carlos S Moreno

**Affiliations:** 1 Department of Pathology & Laboratory Medicine and Winship Cancer Institute, Emory University School of Medicine, Atlanta, GA, USA; 2 Celera Genomics, Rockville, MD, USA; 3 Present address: Microarray Research Facility Section, Research Technologies Branch, NIAID/NIH, Bethesda, MD, USA; 4 Applied Biosystems, Foster City, CA, USA

## Abstract

**Background:**

We previously analyzed human embryonic kidney (HEK) cell lines for the effects that simian virus 40 (SV40) small tumor antigen (ST) has on gene expression using Affymetrix U133 GeneChips. To cross-validate and extend our initial findings, we sought to compare the expression profiles of these cell lines using an alternative microarray platform. METHODS: We have analyzed matched cell lines with and without expression of SV40 ST using an Applied Biosystems (AB) microarray platform that uses single 60-mer oligonucleotides and single-color quantitative chemiluminescence for detection. RESULTS: While we were able to previously identify only 456 genes affected by ST with the Affymetrix platform, we identified 1927 individual genes with the AB platform. Additional technical replicates increased the number of identified genes to 3478 genes and confirmed the changes in 278 (61%) of our original set of 456 genes. Among the 3200 genes newly identified as affected by SV40 ST, we confirmed 20 by QRTPCR including several components of the Wnt, Notch, and Hedgehog signaling pathways, consistent with SV40 ST activation of these developmental pathways. While inhibitors of Notch activation had no effect on cell survival, cyclopamine had a potent killing effect on cells expressing SV40 ST. CONCLUSIONS: These data show that SV40 ST expression alters cell survival pathways to sensitize cells to the killing effect of Hedgehog pathway inhibitors.

## Background

DNA microarray technology enables the investigator to quantitate gene expression of hundreds or thousands of genes from a single RNA sample. However, the various types of commercially available microarray technologies have different genomic targets, use different probes design methodologies, and different detection chemistries making cross-platform comparisons difficult. A number of studies comparing spotted cDNA two-color technology with the Affymetrix single-color technology have found fairly poor correlation of data between these two approaches [1-3]. Other studies comparing oligonucleotide platforms such as Agilent and Affymetrix have found higher correlations [4]. An important difference between Affymetrix GeneChips and other oligonucleotide platforms is that Affymetrix uses multiple 25-mer probe pairs, while all other oligonucleotide microarrays use a single probe per gene, varying in length from 50 to 70 bases.

A relatively new platform for microarray analysis developed by Applied Biosystems (AB) employs single 60-mer oligonucleotides, similar in length to Agilent, but uses single-color chemiluminescence detection technology as opposed to two-color Cy3/Cy5 labeling and laser fluorescence scanning. To compare the AB Expression Array System platform with Affymetrix, we analyzed RNA samples that we had previously analyzed with Affymetrix U133AB GeneChips [5]. Here we show that the AB platform has substantially higher sensitivity, detecting four times as many gene changes in an identical experimental design, and over seven times as many genes when additional technical replicates were included. Moreover, the AB microarray data was well correlated with QRTPCR validation data (R^2 ^= 0.71) while Affymetrix data had lower correlation with QRTPCR results (R^2 ^= 0.47).

In addition, the genes that were identified solely with the AB technology provided insights into the mechanisms by which simian virus 40 small tumor antigen (SV40 ST) affects transformation of human cells that were not apparent in our earlier analyses. We show that SV40 ST induces expression of several key components of the Notch, Wnt, and Hedgehog signaling pathways. While inhibitors of Notch activation had little effect on cell survival, the Hedgehog inhibitor cyclopamine had >50% killing effect on cells expressing SV40 ST, suggesting that SV40 ST makes cells dependent on Hedgehog signaling for survival.

## Methods

### Cell lines

Stable human embryonic (HEK) cell lines HEK-TERST, HEK-TERV have been described previously [5]. Briefly, cells were maintained in α-MEM, 10%FBS, 2 mM/L glutathione, 100 u/ml penicillin/streptomycin. Cells were serum starved in α-MEM, 0.1%FBS, 2 mM/L glutathione, 100 u/ml penicillin/streptomycin for 24 hours prior to preparation of total RNA for microarray analysis.

### Affymetrix genechip expression analysis

Total RNA was prepared from two independent biological replicates of the HEK-TERV and HEK-TERST cell lines and used for whole genome expression analysis as previously described [5]. Data from Affymetrix CEL files was then normalized using the robust multiarray average (RMA) method [6]. Briefly in the original analysis of Affymetrix data, genes called Absent by the Microarray Suite 5.0 software in all hybridizations and genes that were called no change (NC) in more than one ST-TERV Affymetrix comparison file were filtered out leaving 2545 probes for Significance Analysis of Microarrays (SAM) analysis [7]. After data normalization, SAM analysis was performed on the remaining 2545 probe sets using the following relevant parameters: Δ = 0.26, fold-change = 1.5, number permutations = 1000, RNG seed = 1234567, median FDR < 3%, significant probes = 555, predicted false positives = 17. For the revised Affymetrix analysis presented here, all probe sets with at least one present call (n = 15229) were included in the SAM analysis using the following relevant parameters: Δ = 0.59, fold-change = 1.5, number permutations = 500, RNG seed = 1234567, median FDR < 3%, significant probes = 668, predicted false positives = 16.

### AB Expression array system analysis

The quality of the RNA from the samples was evaluated using the Agilent Bioanalyzer 2100 (Agilent Technologies, Palo Alto, CA). A minimum Bioanalyzer RNA Integrity Number (RIN) value of 8 was required prior to RNA labeling. One μg of total RNA from each sample was used to synthesize DIG-labeled cRNA as described by the Applied Biosystems Chemiluminescent RT-IVT Labeling protocol (Applied Biosystems, Foster City, CA). A total of 36 μg of labeled cRNA (18 μg for each process replicate) from each RT-IVT reaction was hybridized onto two Applied Biosystems Human Genome Survey Microarrays following the manufacturer's recommendations. Microarrays were analyzed using the AB1700 Chemiluminescent Microarray Analyzer. Two technical replicates were produced for each biological replicate, for a total of eight hybridizations.

For each gene, the expression values were normalized across arrays by quantile normalization [8]. "Detectable" calls for gene expression were based on Applied Biosystems recommendations (signal/noise ratio >3.0 and FLAG values <5000). A gene had to be called "detectable" in 3 out of 4 replicates in at least one tested sample group (either HEK-TERST or HEK-TERV) to be retained for further analysis. From the initial dataset of 33096 probes, 17710 probes passed this filter and were used in subsequent SAM analyses. Significance analysis using the SAM software was performed on the four hybridizations (excluding technical replicates) using the following relevant parameters: Δ = 0.77, fold-change = 1.5, number permutations = 500, RNG seed = 1234567, median FDR < 3%, significant probes = 1554, predicted false positives = 51. For analysis of all eight hybridizations including technical replicates, SAM analysis was performed with the following parameters: Δ = 0.84, fold-change = 1.5, number permutations = 500, RNG seed = 1234567, median FDR < 3%, significant probes = 3663, predicted false positives = 100. Expression fold-changes between TERST and TERV groups were derived from the mean expression level of a gene in all four replicates. A complete dataset for the AB microarray data has been deposited in the ArrayExpress database, accession number E-MEXP-519. The Affymetrix dataset is available at ArrayExpress, accession number E-MEXP-156.

### Gene network pathway analysis

Gene Ontology (GO) annotations were analyzed using the GOstat software [9,10] and with the Panther Protein Classification System [11] to identify functional annotations that were significantly enriched in this gene set compared to the entire human genome. Gene lists altered by SV40 ST were mapped onto biological pathways that were significantly represented. Lists of significantly changed genes were similarly analyzed using Ingenuity Pathway Analysis [12]. Gene lists were mapped to canonical pathways and relationships were extracted from the Ingenuity Pathways Knowledgebase. Lists were also loaded and analyzed with GenMAPP [13] and genes altered by SV40 ST were analyzed for interaction with the Wnt signaling pathway.

### TaqMan assay validation

Validation of microarray results was done using Applied Biosystems TaqMan Gene Expression Assays [14]. Total RNA from the original samples was converted to cDNA using the Applied Biosystems High Capacity cDNA Archive Kit. Ten ng of cDNA was used per TaqMan reagent based Reaction. Each TaqMan assay was run on an Applied Biosystems 7900HT FAST Real-Time PCR system in quadruplicates and expression fold-changes (normalized to the endogenous control GAPDH) between HEK-TERST and HEK-TERV were derived using the comparative CT method [15]. Concordance of fold-change comparability of selected genes between microarray and TaqMan data was determined by the Pearson correlation coefficient.

### Immunoblots

Immunoblots were performed as previously described [5]. The human activated Notch 2 antibody (Abcam Inc., Cambridge, MA, cat# Ab8926) was derived using a synthetic peptide CRDASNHKRREPVGQD, corresponding to amino acids 1719–1733 of Human activated Notch2 and used at 1:500.

### Promoter analysis

Promoter Analysis was performed using our automated CONFAC software, which identifies promoters sequences within 3 kb upstream of transcription start sites and the first intron of each gene that are conserved between human and mouse genomes [16]. The position weight matrices are those from the MATCH software using the TRANSFAC Professional 8.3 database.

### Drug sensitivity assays

HEKTER-LUK and HEKTER-ST cells were treated with appropriate concentrations of vehicle (Ethanol or DMSO), 2.4μM cyclopamine (C-8700, LC laboratories, Woburn, MA, USA), or 1 nM γ-secretase inhibitor (S-1120, A.G. Scientific, Inc. San Diego, CA, USA). Cells were initially incubated with the compounds for 48 hours and the incubation was extended for another 24 hours with fresh media containing vehicle or drug. Cells were plated at 2 × 10^4^/well in a 96-well plate and viability was determined by the MTT assay. Twenty μl of 3 mg/mL 3- [4,5-dimethylthiazol-2-yl]-2,5-diphenyltetrazolium bromide (MTT) was added to each well and incubated for 4 hours at 37°C. Elution of the precipitate was performed with 100 μL of DMSO and 10μL of Tris-Glycine buffer. Cell viability was calculated from the absorption values obtained at 570 nm using an automated enzyme-linked immunosorbent assay (ELISA) reader. In a separate set of experiments, both HEK-TERV and HEK-TERST cells were plated at 1 × 10^5^/well in a 6 well plate containing α-MEM media (Cellgro Mediatec Inc, Herndon, VA, USA) supplemented with 10% FBS and allowed to attach overnight. Media was then replaced with fresh medium containing either vehicle or drug. Cell viability was determined after 72 hrs by Trypan blue exclusion. Fifty μl of cell suspension was mixed with 50 μl of Trypan blue isotonic solution (0.2%; w/v), and cell viability was determined on a hemocytometer by phase contrast microscopy.

## Results

### Cross-platform microarray analysis

**Figure 1:**
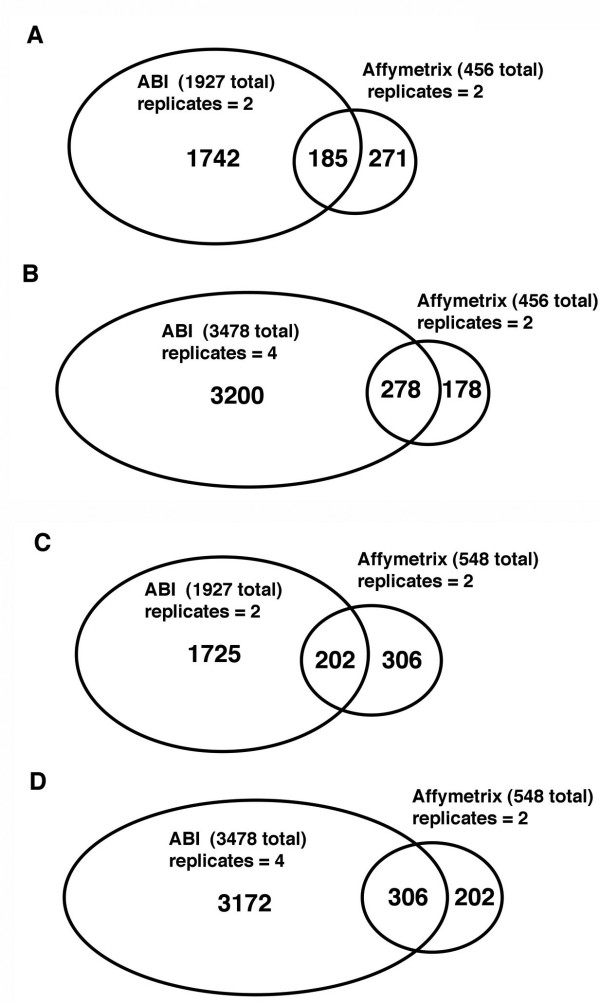
**(A) **Venn diagram of the number of genes identified as changed between HEK-TERST and HEK-TERV cells using the AB and Affymetrix platforms and an equivalent number of replicates. Approximately 40% of previously identified genes were verified with equal replicates and 4.2 times as many genes were significant on the AB platform. **(B) **Venn diagram of the number of genes identified including two additional technical replicates for each cell line. Approximately 61% of previously identified genes were verified with these data, and 7.6 times as many genes were significant on the AB platform. **(C) **Venn diagram of the 548 re-analyzed Affymetrix genes and the 1927 AB genes without technical replicates. **(D) **Venn diagram of the 548 re-analyzed Affymetrix genes and the 3478 AB genes with technical replicates.

We assessed the cross-platform comparability and performance of the Affymetrix U133AB GeneChip with the AB 1700 Chemiluminescent Microarray Analyzer using RNA prepared from HEK-TERV cells that do not express SV40 ST and HEK-TERST cells that do express SV40 ST. Two biological replicates were analyzed on each array platform, and in addition, two technical replicates were performed on the AB platform. To make a fair comparison of the sensitivity of the two systems, we analyzed the AB data without the technical replicates so that equal numbers of hybridizations were used for analysis of each technology. Significantly changed genes were identified using identical parameters of 1.5 fold change and a false discovery rate of 3% using SAM analysis [7]. Figure 1A shows a Venn diagram of the number of genes altered by expression of SV40 ST that were detected by each platform alone or by both platforms. Figure 1B shows a Venn diagram of the number of genes in common and detected when an additional set of AB technical replicates are included in the analysis. Previously, we identified 456 genes whose expression was significantly increased/decreased due to ST expression on the Affymetrix platform [5]. The AB platform identified 2014 significantly different probe sets corresponding to 1927 unique genes using identical replicates and analysis techniques. The total number of genes identified by both platforms was 185 or 41% of the 456 genes initially identified with the U133 GeneChips. When technical replicates were included in the AB analysis, 3478 unique genes were identified as affected by SV40 ST [see Additional file 1], increasing the number of confirmed genes from our previous analysis to 278 genes (61%) from the original set of 456 genes. Of those 456 genes, ten corresponded to EST sequences that had no equivalent probes on the AB microarrays, so at most 446 genes could have been confirmed.

Because our original SAM analysis of our Affymetrix data was performed on a prefiltered set of only 2545 probe sets, we performed another SAM analysis on a larger set of Affymetrix probes. For the revised SAM analysis, we included all of the 15,229 Affymetrix probe sets with a present call in at least one sample. Despite the larger number of probe sets included in the analysis, the number of significant genes identified increased by only 92 genes for a total of 548 significant genes [see Additional file 2]. Of those 548 genes, 202 (37%) overlapped with the 1927 AB genes identified without technical replicates (Figure 1C). When compared to the 3478 genes identified on the AB platform with technical replicates, there were 306 genes (56%) that overlapped (Figure 1D). These data show that the AB platform was able to measure significant changes in many more genes than the Affymetrix platform and that additional technical replicates nearly doubled the number of detectable differences. A total of 178 genes called significant by the Affymetrix platform were not confirmed using the AB1700 [see Additional file 3].

**Figure 2:**
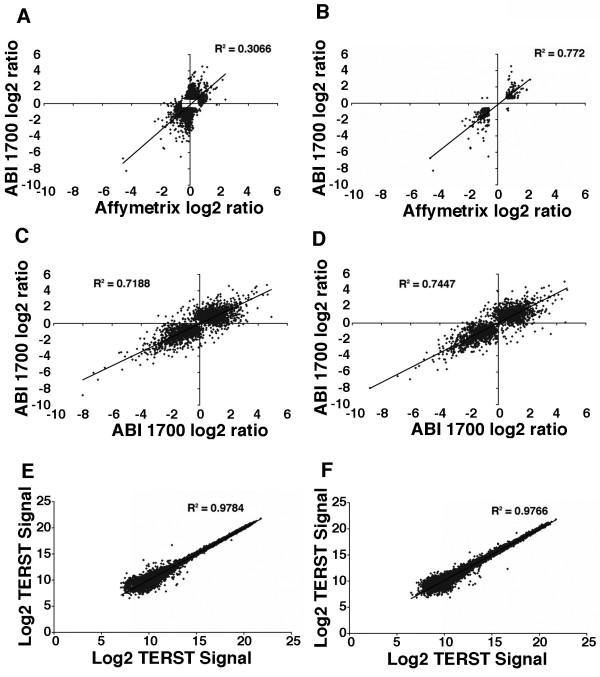
**(A) **Scatterplot comparison of the log2 ratio of gene expression data between HEK-TERST and HEK-TERV cells. Log2 ratios from the AB platform are plotted on the y-axis and from the Affymetrix platform on the x-axis. The genes plotted include any gene identified as significantly changed on either platform. **(B) **Scatterplot comparison similar to panel (A), except that only the 278 genes significantly altered on both platforms are shown. **(C) **Scatterplot of the log2 ratio of HEK-TERST and HEK-TERV cells comparing different technical replicates on the AB platform. **(D) **Scatterplot comparison as in panel (C), except comparing biological replicates on the AB platform. **(E) **Scatterplot comparison of the log2 transformed absolute signal from HEK-TERST cells from two technical replicates on the AB platform. **(F) **Scatterplot comparison of the log2 transformed absolute signal from HEK-TERST cells from two biological replicates on the AB platform.

To directly compare the expression data from the two microarray platforms, scatter plots of the log2 change between the TERST and TERV cell lines were generated for two datasets. The first dataset included any of the 3666 genes that were identified as significant on either platform (Figure 2A), while the second dataset included only the 278 genes that were significantly changed on both platforms (Figure 2B). As can be seen, the correlation for the genes found by both platforms is quite high (R^2 ^> 0.77), and consequently the confidence for the differential expression of these genes is also very strong. A complete list of these 278 genes is included in the Supplementary Data [see Additional file 4], and it includes many genes that we characterized previously such as myc, CD24, PAR2/F2R, PAR3/F2RL1, PAR4/F2RL2, IL-8, IQGAP2, ICAM1, and HOXB6. For comparison, the reproducibility of biological (R^2 ^> 0.71) and technical (R^2 ^> 0.74) replicates on the AB platform is also shown for the 3478 genes with significant changes (Figures 2C and 2D). Also shown are the scatterplots for absolute signal in technical and biological replicates (R^2 ^> 0.97) for the HEK-TERST cell line using the AB platform (Figures 2E and 2F). These data show that the AB platform data are highly reproducible.

The difference in the range of the data from the two expression platforms in Figures 2A and 2B is worth noting. In general, the dynamic range of the Affymetrix data was smaller (from 2^-5 ^to 2^3^) than the AB dynamic range (from 2^-9 ^to 2^5^). This may account for the greater number of genes that were significantly altered on the AB platform due to the nature of chemiluminescence with higher signal range and lower background. In addition, the potential for greater sensitivity of 60-mer oligonucleotides compared to 25-mer oligonucleotides, as well as the sequence specificity of the 60-mers may also account for the greater number of genes that were significantly altered on the AB platform.

### QRTPCR taqman validation

**Figure 3:**
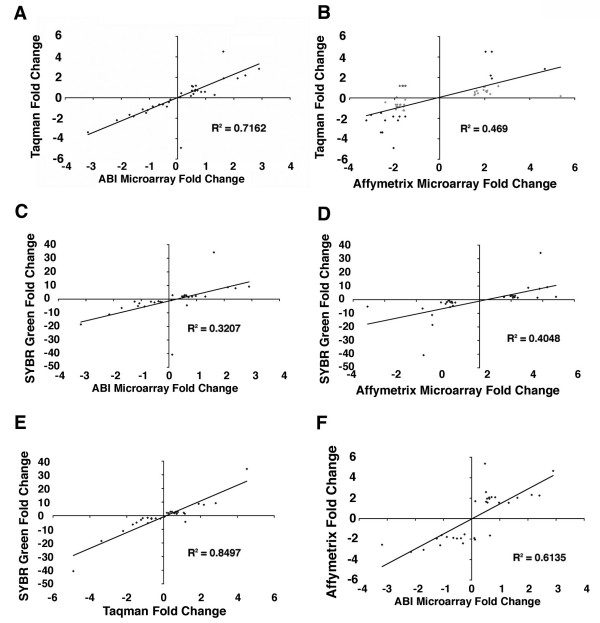
**(A) **QRTPCR validation of the AB Expression Array System measured Fold Change of gene expression data between HEK-TERST and HEK-TERV cells. Fold change values from the AB platform are plotted on the x-axis and TaqMan assay based QRTPCR fold change is plotted on the y-axis. Data for the same set of genes are shown in all panels (A-F). **(B) **QRTPCR validation of the Affymetrix measured Fold Change of gene expression data between HEK-TERST and HEK-TERV cells. Fold change values from the Affymetrix platform are plotted on the x-axis and TaqMan assay based QRTPCR fold change is plotted on the y-axis. **(C) **Comparison of SYBR Green reagent based QRTPCR data to the AB Expression Array System fold change data. **(D) **Comparison of SYBR Green regent based QRTPCR data to the Affymetrix fold change data. **(E) **Comparison of the SYBR Green regent based QRTPCR data and the TaqMan assay based QRTPCR data. **(F) **Scatterplot comparison of the Affymetrix measured and AB Expression Array System measured Fold Change of gene expression data between HEK-TERST and HEK-TERV cells.

As an independent method to determine the accuracy of expression changes on the two platforms, we chose to perform TaqMan assay based QRTPCR on 49 genes that we previously determined were altered by SV40 ST using Affymetrix arrays. Figure 3A shows the correlation of the QRTPCR TaqMan assay based data and the AB microarray data was excellent (R^2 ^> 0.71). In contrast, the correlation of the Affymetrix data with the same TaqMan assay data was fairly low (R^2 ^~0.47, Figure 3B). Moreover, only 22 of the 49 genes (45%) were confirmed by TaqMan assay based QRTPCR. These data contrast sharply with our earlier data in which 43 of 46 genes (93%) were confirmed using SYBR Green reagent based QRTPCR to quantitate mRNA changes [5]. To resolve this conflict, we plotted our earlier SYBR Green reagent based QRTPCR against the AB microarray data (Figure 3C) and the Affymetrix data (Figure 3D). The large difference in the scales of the TaqMan data and the SYBR Green reagent data indicate that the SYBR Green regent based QRTPCR overestimates changes in gene expression. Nevertheless, the SYBR Green and TaqMan assay based QRTPCR data were very well correlated (Figure 3E), demonstrating that the discrepancy is largely a matter of sensitivity and signal amplification. Interestingly, the Affymetrix data was better correlated with the AB microarray data than it was with either of the QRTPCR data sets (Figure 3F). This may be due to the fundamental differences between microarray hybridization data and quantitative real-time PCR.

To validate the 3200 genes that were uniquely identified with the AB technology, we selected an additional 23 genes for TaqMan assay based QRTPCR validation (Table 1). The validation data for these genes was equally well correlated with the AB microarray data (R^2 ^> 0.71). A complete list of the 3478 genes identified with the AB platform is given in the Supplementary Data [see Additional file 1].

### Pathway annotation of ST-regulated genes

We analyzed the Gene Ontology (GO) annotation of the 3478 genes identified with the AB Microarray using the GOstat software [9] and identified functional annotations that were significantly enriched in this gene set compared to the entire human genome. The significant GO categories are summarized in Table 2 and parallel those categories we previously observed [5]. We also analyzed the same gene set with the Panther Protein Classification System [11] and obtained similar results shown in Table 3.

### SV40 ST may increase notch signaling

Among the genes we observed to be upregulated by SV40 ST were Delta-like ligand 1 (DLL1, up 5.3 fold), a human homolog of the *D. melanogaster *ligand *delta*, and Jagged 1 (JAG1, up 1.6 fold) a homolog of the Notch ligand *serrate*. To determine whether the Notch receptor was expressed in HEK-TERST cells, we examined the expression profiling data from both our Affymetrix and AB expression studies. Data from both platforms indicated that while the Notch 1, Notch 3, and Notch 4 homologs had very low expression levels, the Notch 2 receptor was highly expressed in both the HEK-TERV and the HEK-TERST cell lines. Moreover, both platforms indicated that there was little if any change in Notch 2 expression between the HEK-TERV and HEK-TERST cells.

In a recent study [17], mesothelial cells required both SV40 ST and SV40 LT for induction of Notch receptor expression, and required activation of the MAPK-ERK pathway. Since the HEK-TERV cells express both SV40 LT and mutant H-Ras-V12, they have constitutive activation of the MAPK-ERK pathway even in the absence of SV40 ST. Thus, our data are consistent with the observation that LT and ERK activation are sufficient to activate Notch expression. However, the mesothelial cells also constitutively expressed DLL1 and JAG1 ligands with or without expression of SV40 ST. Thus, in HEK cells, it appears that ST increases DLL1 and JAG1 expression, which are already expressed in mesothelial cells. In light of these data, we predict that enhanced expression of both DLL1 and Notch2 on the HEK-TERST cells would increase activation of Notch signaling via cell-cell interactions between adjacent HEK-TERST cells.

**Figure 4:**
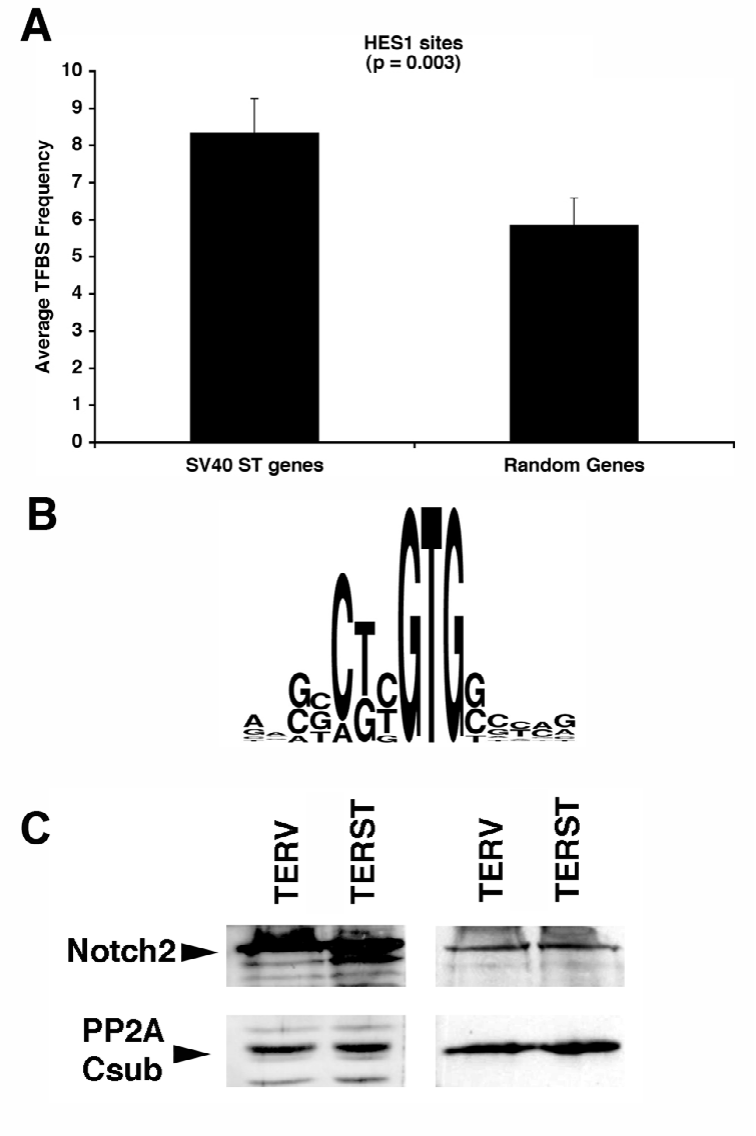
**(A) **Plot of the average frequency of HES1 sites in genes upregulated by ST > 2-fold and in a random control list of 200 genes. **(B) **Sequence logo of the position weight matrix for HES1 sites based on the TRANSFAC database.**(C) **Immunoblot of whole cell lysates prepared from HEK-TERST and HEK-TERV cells and probed with antibodies specific for the activated human Notch-2. Activated Notch-2 is equally present in both cell lines (right), although longer exposures (left) reveal a shorter isoform that is more abundant in cells expressing SV40 ST.

We next examined the promoters of the genes altered by ST to determine if they were consistent with activated Notch signaling. Using our CONFAC software [16], we compared the promoters of 256 RefSeq genes upregulated by ST (> 2-fold increase and FDR < 1%) with sets of random promoters. Since Notch signals are mediated through Hairy-enhancer of split 1 (HES1) [18], we predicted that if Notch signaling played an important role in regulation of the genes affected by ST, the promoters of these genes should be enriched for HES1 sites. Our CONFAC analysis of these 256 upregulated RefSeq genes found that 106 genes contained conserved transcription factor binding sites (TFBS) and that HES1 sites were the single most significantly enriched TFBS (p = 0.003) in the promoters of these 106 genes (Figure 4A). Of these 106 genes, 91 contained conserved HES1 sites. The ST-upregulated genes containing multiple conserved HES1 sites included DLL1, the c-myc oncogene, inhibitor of DNA binding 2 (ID2), frizzled-related protein (FRZB), Dickkopf homolog 1 (DKK1), and hedgehog-interacting protein (HIP). A complete list of these 106 genes and the number of conserved HES1 sites in their proximal promoters is given in the Supplementary Data [see Additional file 5] and the HES1 site used for this analysis is shown in Figure 4B.

To directly test whether Notch-2 is activated in HEK-TERST cells, we probed immunoblots of whole cell lysates from HEK-TERV and HEK-TERST cells using polyclonal antibodies specific to activated human Notch-2 (Figure 4C). Notch is translated as a ~300 kD full length preprotein (Notch-FL) that is proteolytically cleaved to produce a mature ~100 kD transmembrane/cytosolic C-terminal fragment (Notch-TM) and an extracellular N-terminal fragment [19]. Upon binding of the extracellular fragment to the DLL1 ligand, the Notch-TM is cleaved by gamma-secretase [20,21] to produce the intracellular fragment (Notch-IC) that translocates to the nucleus to activate transcription [22]. Short exposures of Activated Notch-2 immunoblots indicate no change in activation of Notch-2 between HEK-TERV and HEK-TERST cells, but longer exposures demonstrated a shorter isoform of Notch-2 present in HEK-TERST cells that is not present in HEK-TERV cells (Figure 4C). Thus, while there may be increased Notch signaling in cells expressing SV40 ST, it is clear that Notch-2 is also activated in the HEK-TERV cells that do not express SV40 ST.

### SV40 ST also activates components of the Wnt and Hedgehog pathways

**Figure 5:**
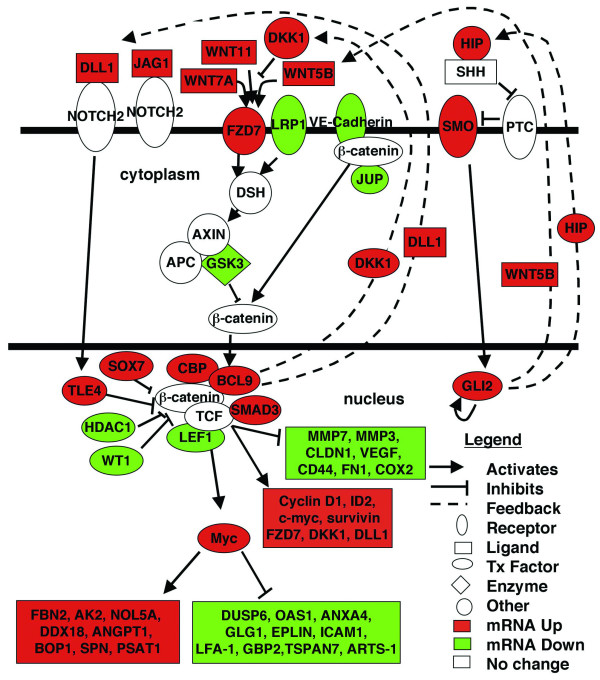
Impact of SV40 ST on the Notch, Wnt, and Hedgehog Pathways. Schematics of the Notch, Wnt, and Hedgehog pathways are shown. Genes with significantly increased mRNA's in HEK-TERST cells compared to HEK-TERV cells are shown in red, while genes significantly decreased are shown in green. Sets of downstream targets of the Wnt pathway are grouped in boxes, as are sets of c-myc downstream targets. Activation of the Hedgehog pathway due to increased HIP, SMO, and GLI2 levels results in increased Wnt ligand expression. Activation of Wnt signaling increases c-myc, activating and repressing multiple downstream target genes. Wnt pathway activation also feeds back to activate expression of Notch ligands such as DLL1.

Among the genes upregulated by ST were several components of the Wnt and Hedgehog pathways (Figure 5), including the WNT5B ligand (up 2 fold). We also detected increased levels of the Smoothened receptor (1.9 fold) and the GLI2 transcription factor (2.2 fold), both mediators of Hedgehog signaling, as well as the hedgehog antagonist HIP (3.9 fold). Smoothened expression is essential for Hedgehog signaling [23], and three Hedgehog target genes, GLI2 [24], HIP [25], and Wnt5A [26], were upregulated, suggesting that Hedgehog pathways are activated by SV40 ST and that they contribute to activation of Wnt signaling. SV40 ST also increased expression of the Wnt antagonist DKK1 (up 5 fold) that was recently shown to be a direct target of the Wnt-β-catenin/TCF pathway [27]. While DKK1 was not identified in our original analysis of the Affymetrix data, it was one of the new 92 genes that were significantly changed in the revised analysis of the Affymetrix data. In addition, the Wilm's Tumor suppressor (WT1) gene, which when lost in Wilm's tumors can result in activation of β-catenin [28], was downregulated 8-fold in HEK-TERST cells. Another member of the Wnt pathway, transducin-like enhancer of split 4 (TLE4), which is a homolog of the *groucho *repressor of the Wnt pathway and a target of Notch signaling, was increased 1.7 fold by SV40 ST. Three Wnt ligands (WNT5B, WNT11, and WNT7A), one Wnt receptor (FZD7), and at least fourteen known Wnt downstream targets [29], including c-myc, DKK1, and DLL1 were all affected by SV40 ST expression (Figure 5). Thus, the altered levels of multiple Wnt pathway components and Wnt target genes strongly suggest that SV40 ST activates Wnt signaling.

### Hedgehog inhibitors, but not Notch inhibitors, kill cells expressing SV40 ST

**Figure 6:**
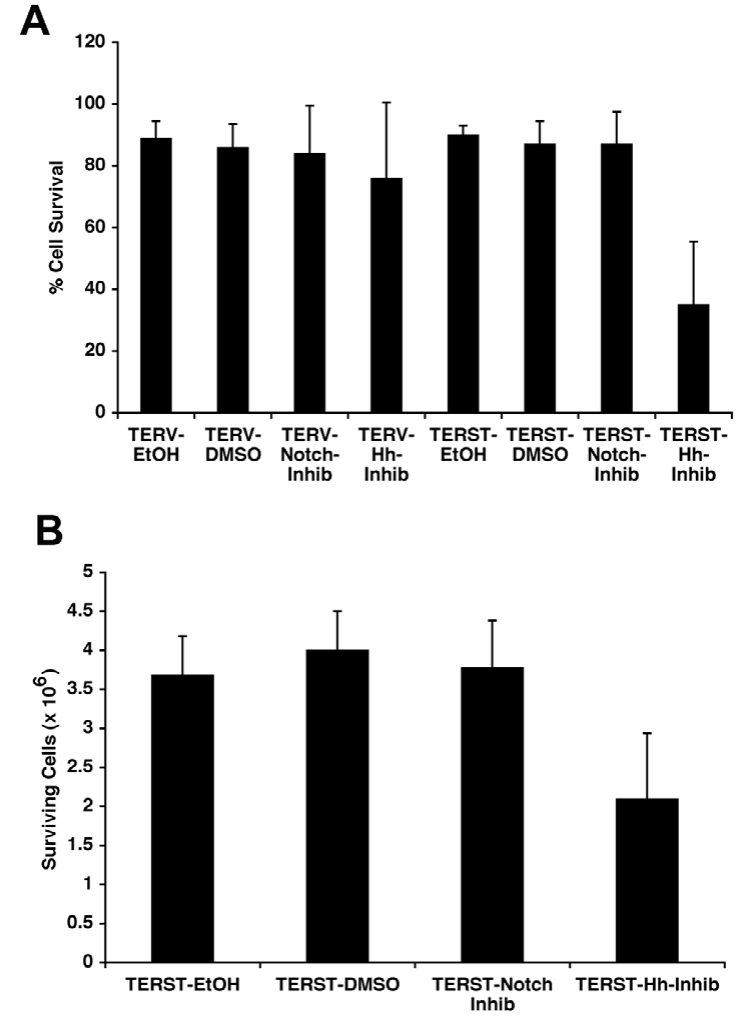
Antitumor activities of cyclopamine and γ-secretase inhibitor against HEKTER-LUK and HEKTER-ST cell lines. **(A) **Survival of HEK-TERV and HEK-TERST cells determined by MTT assay after treatment with vehicle, 2.4μM Cyclopamine or 1 nM γ-secretase inhibitor for 72 hrs. Mean and standard error are shown for each treatment. Percent cell survival was computed relative to untreated cells. **(B) **Survival of HEK-TERST cells determined by Trypan Blue exclusion assay after treatment of 1 × 10^5 ^cells with vehicle, 2.4μM Cyclopamine or 1 nM γ-secretase inhibitor for 72 hrs.

To directly test the importance of the activation of the Notch and Hedgehog pathways for survival of cells expressing SV40 ST, we treated HEK-TERV and HEK-TERST cells with inhibitors of the Notch-activating protease, gamma-secretase, and the Hedgehog-activated receptor, Smoothened. While the gamma-secretase inhibitor had little effect on survival of HEK-TERV or HEK-TERST cells, the Smoothened-inhibitor, cyclopamine, had a marked killing effect on HEK-TERST cells (Figure 6). Moreover, the SV40 ST expressing cells were significantly more sensitive to cyclopamine's killing effects, suggesting that SV40 ST sensitizes cells by making them dependent on pro-survival signals from the hedgehog pathway.

## Discussion

We have used a relatively new commercial DNA microarray platform to analyze the genes that are affected by expression of SV40 ST in HEK cells and to compare the performance of two single-color, oligonucleotide-based technologies. In general, the AB microarray platform outperformed the Affymetrix platform in both sensitivity and accuracy. The AB platform identified over 3.5 times as many genes (1927 vs. 548) using equivalent replicates and analyses, and over six times as many genes (3478 vs. 548) with additional technical replicates. The AB data was highly reproducible, had a greater dynamic range than the Affymetrix data, and correlated much better with TaqMan assay based QRTPCR validation data in our experiments.

The fact that the AB microarray uses 60-mer oligonucleotides compared to the 25-mer probe sets used in Affymetrix microarrays likely contributed greatly to the differences in specificity, sensitivity, and accuracy. Apart from the difference in the length of the oligonucleotides, the actual sequences that were queried were different between the two platforms, making cross-platform annotation somewhat difficult. The TaqMan assay based QRTPCR assays used were not designed biased to any particular region of each target transcript, while the probes on the AB microarray were biased to the 3' UTR (within 1500 bp of the 3' UTR), making these assays independent for mRNA quantitation. However, each probe on the AB microarray queries sequences in common to all verified alternative splice forms of each gene, while the TaqMan assays can measure a subset of specific splice forms. Thus, in the few instances in which the TaqMan and AB microarray data did not agree, it is likely to be either a function of higher sensitivity of TaqMan or that the TaqMan assays are targeting a subset of spliced transcripts that the microarrays measures. Moreover, it is entirely possible that the Affymetrix and AB platforms can be measuring alternative splice forms of the same gene.

The classes of genes that were altered based on GO ontologies were in general the same as that observed previously in our original analysis [5]. Specifically, we once again observed large decreases in expression of immune response genes, and changes in genes involved in cell cycle regulation, oncogenesis, cellular adhesion, and signal transduction. However, the statistical significance of these observations was increased due to the greater number of genes detected. For example, we found 70 genes annotated for wounding response were affected (p = 9.63E-12), while our earlier study found 48 genes (p = 3.46E-4). In addition, a number of new, biologically significant observations became apparent from the AB expression data that we did not observe previously.

**Figure 7:**
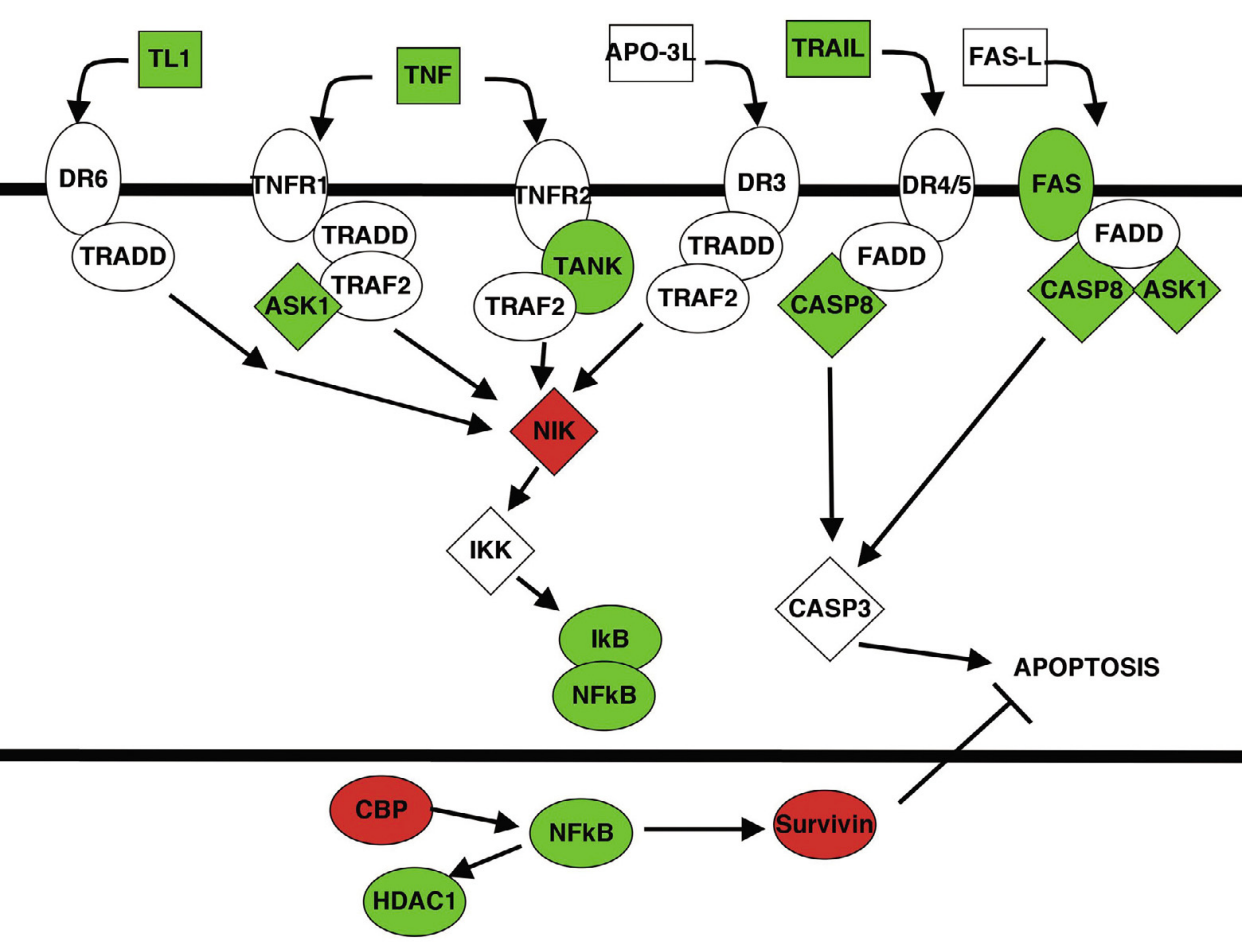
Effect of SV40 ST on Death Receptor Pathways. The diagram is colored as in Figure 5. SV40 ST downregulates pro-apoptotic components of all death receptor pathways and upregulates anti-apoptotic components such as survivin.

Our initial analysis with Affymetrix arrays identified downregulation of interleukin 8 (IL-8) and interleukin 1β (IL-1β). With the AB platform, we also observed downregulation of interleukin 1α (IL-1α), interleukin 1 receptor (IL1R), interleukin 6 (IL-6) as well as the chemokine ligands CXCL2, CXCL3, CXCL5, CCL20, and tumor necrosis factor (TNF). In addition, several components of the TNF-NFκB signal transduction cascade were downregulated, including the TNF receptor associated 5 (TRAF5) protein, toll-like receptor 2 (TLR2), receptor-interacting protein kinase 2 (RIPK2), interleukin-1 receptor-associated kinase 2 (IRAK2), NFKB1, REL, Caspase 8 (CASP8), and TNF-related apoptosis inducing ligand (TRAIL/TNFSF10). The strong downregulation of multiple components along the TNF-NFκB pathway explains why so many inflammatory genes are downregulated by SV40 ST. Furthermore, we observed changes in components of every known death receptor pathway (Figure 7), including FAS, TNF, CASP8, TRAIL, TL1 [30], and apoptosis signal-regulating kinase 1 (ASK1) [31], thus providing mechanisms for SV40 ST induced resistance to apoptosis.

In our earlier study [5], we also determined that cell-cell adhesion and integrin signaling were altered in cells that express SV40 ST. The larger dataset of altered genes from the AB platform provides additional insights into these changes. In addition to our earlier observations on ICAM1, VCAM1, junction plakoglobin (JUP), proto-cadherins, collagens, and laminins, the AB expression data showed decreases in integrins α_2_, α_3_, α_V_, α_X_, and β_8 _and increases in integrins α_4 _and α_7_. Integrins α_3_, α_4 _and α_7 _mediate binding to laminins, while α_2 _integrin binds to collagen, and integrin α_Vβ8 _is an RGD receptor (reviewed in [32]). We also observed alterations in both directions for seven laminin genes and seven collagen genes, suggesting that, in addition to modulating cellular adhesion in favor of laminin-binding receptors, SV40 ST is altering the composition of the extracellular matrix. The AB data also provided additional evidence for decreased cell-cell adhesion through downregulation of neural cell adhesion molecule 1 (NCAM1), L1 cell adhesion molecule (L1CAM), claudin 1 (CLDN1), and junction adhesion molecule 2 (JAM2).

Regarding cellular proliferation, we had previously reported increased levels of myc and src activation. The additional expression data described here show increased levels of epidermal growth factor receptor (EGFR) and insulin-like growth factor 2 (IGF2), providing potential mechanisms by which growth-factor signaling cascades can be stimulated by SV40 ST. Moreover, we observed activation of the Wnt signaling pathway, providing another mechanism that may explain the observed increases in myc expression, since myc is a known downstream target of β-catenin [33] and Wnt signaling provides resistance to myc-induced apoptosis [34]. We also observed increased expression of the Wnt5b ligand, as well as DKK1, a recently validated downstream target of Wnt signaling [27]. At least fourteen known Wnt downstream targets [29] were affected by SV40 ST, further supporting the evidence that Wnt signaling is activated by SV40 ST.

The observation that cyclopamine killed 50–60% of HEK-TERST cells, but had little effect on HEK-TERV cells suggests that SV40 ST exerts changes within the cell that makes it dependent on hedgehog signals for survival. However, the lack of an effect of gamma-secretase inhibitors suggests that Notch signaling is not essential for survival in cells expressing SV40 ST.

## Conclusion

Our data show that the AB platform had substantially higher sensitivity than the Affymetrix platform in these experiments, detecting four times as many gene changes. Moreover, the AB data was highly correlated with QRTPCR validation data (R^2 ^= 0.71) while Affymetrix data had lower correlation with QRTPCR results (R^2 ^= 0.47).

Among the Wnt target genes that were upregulated by SV40 ST, we observed changes in key developmental regulators, including the DLL1 and JAG1 ligands for Notch. However, activated Notch-2 was detected in both cell lines, suggesting that its activation, while possibly increased by SV40 ST, was not dependent on SV40 ST. Finally, the increased levels of the Smoothened receptor (SMO) and three Hedgehog target genes (GLI2, HIP, and Wnt5A), suggest that Hedgehog pathways are also activated by SV40 ST, and the cell death induced by cyclopamine shows that Hedgehog activation is critical to survival for cells expressing SV40 ST. These data support a model in which Hedgehog signaling activates Wnt signaling, which in turn activates Notch signaling (Figure 5). The activation of all three of these developmental pathways provides a mechanism for our earlier conclusions [5] that SV40 ST induces a de-differentiated phenotype in transformed human cells. Together, the changes in adhesion, proliferation, apoptosis, and differentiation orchestrated by SV40 ST provide several insights into the pathways that are disrupted upon transformation of human cells.

## Abbreviations

SV40 ST: Simian Virus 40 Small Tumor Antigen, AB: Applied Biosystems, RNG: Random number generator, FDR: False Discovery Rate, RT-IVT: Reverse Transcription-In Vitro Transcription.

## Competing interests

The author(s) declare that they have no competing interests.

## Pre-publication history

The pre-publication history for this paper can be accessed here:



## Supplementary Material

Additional file 1 Supplementary Table 1 – excel spreadsheet – TableS1.xls All 3478 genes identified as changed by SV40 ST on the AB1700 Microarray Click here for file

Additional file 2 Supplementary Table 2 – excel spreadsheet – TableS2.xls Genes significantly altered by SAM analysis using 15229 Affymetrix probe sets (q < 3%, fold change > 1.5) Click here for file

Additional file 3 Supplementary Table 3 – excel spreadsheet – TableS3.xls Genes identified as significant by Affymetrix, but not confirmed by AB1700 Click here for file

Additional file 4 Supplementary Table 4 – excel spreadsheet – TableS4.xls Genes significantly altered by SAM analysis on both AB and Affymetrix platforms (q < 3%, fold change > 1.5) Click here for file

Additional file 5 Supplementary Table 5 – excel spreadsheet – TableS5.xls Conserved HES1 sites in promoters of ST upregulated genes Click here for file

## Figures and Tables

**Table 1:** TaqMan assay based QRTPCR and Microarray Fold changes for 23 genes. Genes that were not significant on the Affymetrix column are indicated (n.s.). Twenty genes were validated and three showed little change (BIRC5, WNT7A, and ETS1).

*Gene*	*Expression Array SystemFold Change*	*TaqMan Fold Change*	*Affymetrix Fold Change*
DLL1	5.3	16.3	n.s.
SERPINB3	4.8	10.2	n.s.
NTRK2	6.9	6.5	3.21
STC2	6.9	5.7	n.s.
DKK1	5.1	5.1	1.54
CLDN2	4.8	3.8	n.s.
AK5	3.2	3.8	3.98
GLI2	2.2	2.5	n.s.
MID1	2.1	2.0	n.s.
WNT5B	2.0	1.9	n.s.
GADD45B	2.2	1.8	n.s.
HOXB6	1.6	1.7	2.11
BIRC5	1.5	1.1	n.s.
WNT7A	1.7	1.1	n.s.
ETS1	2.5	1.1	1.54
GFI1	-6.7	-8.6	n.s.
CXCL3	-6.8	-9.2	n.s.
CLDN4	-6.9	-11.9	n.s.
NCAM1	-6.7	17.4	n.s.
WT1	-7.5	-19.3	n.s.
CXCL5	12.2	-32.6	n.s.
GATA3	-8.8	-126.2	n.s.
NOX5|SPESP1	-10.1	-2193.6	n.s.

**Table 2:** GOStat analysis of Biological Processes that are significantly enriched in the set of 3478 genes found with the AB platform. Of those genes only 2476 had recognizable HUGO symbols and 1811 were annotated in the GO database. Shown here is a subset of 20 representative significant GO annotations. The complete set of 135 significant GO categories together with the identities of the genes in each category is included in the supplementary data.

*GO Number*	*GO Category*	*Observed Genes*	*Total Genes*	*p-value*
GO:0007155	cell adhesion	129	859	2.96E-18
GO:0009611	response to wounding	70	429	9.63E-12
GO:0008283	cell proliferation	78	526	2.69E-10
GO:0006954	inflammatory response	43	225	2.80E-10
GO:0009613	response to pathogen	79	542	3.94E-10
GO:0007154	cell communication	447	4919	1.89E-09
GO:0006950	response to stress	137	1164	3.90E-09
GO:0009653	morphogenesis	145	1307	1.12E-07
GO:0006928	cell motility	35	211	4.47E-06
GO:0006935	chemotaxis	24	124	7.56E-06
GO:0009887	organogenesis	112	1026	1.40E-05
GO:0005578	extracellular matrix	58	453	4.10E-05
GO:0000074	regulation of cell cycle	60	483	8.49E-05
GO:0007243	protein kinase cascade	38	283	0.001
GO:0043123	positive regulation of I-kappaB kinase/NF-kappaB cascade	17	91	0.001
GO:0008219	cell death	65	595	0.004
GO:0005911	intercellular junction	19	116	0.004
GO:0000165	MAPKKK cascade	16	92	0.005
GO:0001525	angiogenesis	11	53	0.02
GO:0007219	Notch signaling pathway	5	15	0.05

**Table 3:** Significantly overrepresented GO categories from the Panther annotation database using the same gene set as Table 1.

*Biological Process*	*Observed Genes*	*Expected Genes*	*p-value*
Cell structure and motility	175	118	3.6E-07
Cell communication	207	150	3.4E-06
Protein glycosylation	41	19	4.1E-06
Signal transduction	512	424	4.5E-06
Cell adhesion	107	68	5.6E-06
Carbohydrate metabolism	96	60	9.6E-06
Immunity and defense	220	166	2.5E-05
Protein modification	164	120	6.4E-05
Cell proliferation and differentiation	127	89	8.1E-05
Cell cycle	141	101	8.3E-05
Oncogenesis	101	69	1.6E-04
Cell structure	106	73	1.6E-04
Protein metabolism and modification	381	319	1.9E-04
Cell cycle control	75	49	2.3E-04
Developmental processes	304	249	2.4E-04
Extracellular matrix protein-mediated signaling	17	6	2.7E-04
Intracellular signaling cascade	138	103	4.1E-04
Tumor suppressor	26	13	0.001
Other polysaccharide metabolism	27	14	0.001
Apoptosis	81	57	0.001
Other metabolism	90	65	0.002
Receptor protein tyrosine kinase signaling pathway	36	21	0.002
Skeletal development	26	14	0.002
NF-kappaB cascade	18	8	0.003
Cell motility	52	35	0.003
Nucleoside, nucleotide and nucleic acid metabolism	441	390	0.004
Cell adhesion-mediated signaling	52	35	0.004
Mesoderm development	83	62	0.005
DNA replication	20	11	0.007
DNA metabolism	53	37	0.007
Lipid, fatty acid and steroid metabolism	105	82	0.008
Stress response	35	22	0.008
